# Mechanisms and functions of SUMOylation in health and disease: a review focusing on immune cells

**DOI:** 10.1186/s12929-024-01003-y

**Published:** 2024-01-27

**Authors:** Chien-Hsin Huang, Tsan-Tzu Yang, Kuo-I Lin

**Affiliations:** 1 Genomics Research Center, Academia Sinica, 128 Academia Road, Sec. 2, Nankang District, Taipei, 115 Taiwan; 2 Graduate Institute of Immunology, College of Medicine, National Taiwan University, Taipei, 110 Taiwan

**Keywords:** SUMOylation, Ubc9, SENPs, Immune cells

## Abstract

SUMOylation, which is a type of post-translational modification that involves covalent conjugation of small ubiquitin-like modifier (SUMO) proteins to target substrates, regulates various important molecular and cellular processes, including transcription, the cell cycle, cell signaling, and DNA synthesis and repair. Newly synthesized SUMO is immature and cleaved by the SUMO-specific protease family, resulting in exposure of the C-terminal Gly–Gly motif to become the mature form. In the presence of ATP, mature SUMO is conjugated with the activating enzyme E1 through the cysteine residue of E1, followed by transfer to the cysteine residue of E2-conjugating enzyme Ubc9 in humans that recognizes and modifies the lysine residue of a substrate protein. E3 SUMO ligases promote SUMOylation. SUMOylation is a reversible modification and mediated by SUMO-specific proteases. Cumulative studies have indicated that SUMOylation affects the functions of protein substrates in various manners, including cellular localization and protein stability. Gene knockout studies in mice have revealed that several SUMO cycling machinery proteins are crucial for the development and differentiation of various cell lineages, including immune cells. Aberrant SUMOylation has been implicated in several types of diseases, including cancers, cardiovascular diseases, and autoimmune diseases. This review summarizes the biochemistry of SUMO modification and the general biological functions of proteins involved in SUMOylation. In particular, this review focuses on the molecular mechanisms by which SUMOylation regulates the development, maturation, and functions of immune cells, including T, B, dendritic, and myeloid cells. This review also discusses the underlying relevance of disruption of SUMO cycling and site-specific interruption of SUMOylation on target proteins in immune cells in diseases, including cancers and infectious diseases.

## Background of SUMOylation

### Mechanisms of SUMOylation

SUMOylation is a dynamic and reversible process of post-translational modification (PTM). This modification involves several proteins, including small ubiquitin-like modifier (SUMO), enzymes that catalyze the conjugation, and enzymes that remove conjugated SUMO from substrates. SUMO has 18% amino acid sequence similarity to ubiquitin and plays a critical role in the biology of most eukaryotic organisms [1–3]. This PTM affects various aspects of protein functions, including stability, localization, and transcriptional regulation, which have a significant effect on cellular processes and contribute to both physiological and pathophysiological states in health and disease [4].

The SUMO protein family consists of five paralogs in mammals: SUMO1, SUMO2, SUMO3, SUMO4, and SUMO5, each of which is 10–20 kDa in size [5–12]. SUMO2 and SUMO3 are highly similar with 95% sequence identity, but have only 45% similarity to SUMO1 [13]. SUMO4 has similarity of 86% to SUMO2/3 [6, 14]. Although the paralogs share some identity, many questions related to whether they function redundantly have been raised. Previous reports have shown that SUMO paralogs have different localizations in cells, suggesting that they may function differently [15]. SUMO2/3 are found in nucleoplasm and promyelocytic leukemia protein (PML) bodies. SUMO1 is found not only in nucleoplasm and PML bodies but also in nucleoli, the nuclear envelope, and cytoplasmic foci [15].

**Fig. 1:**
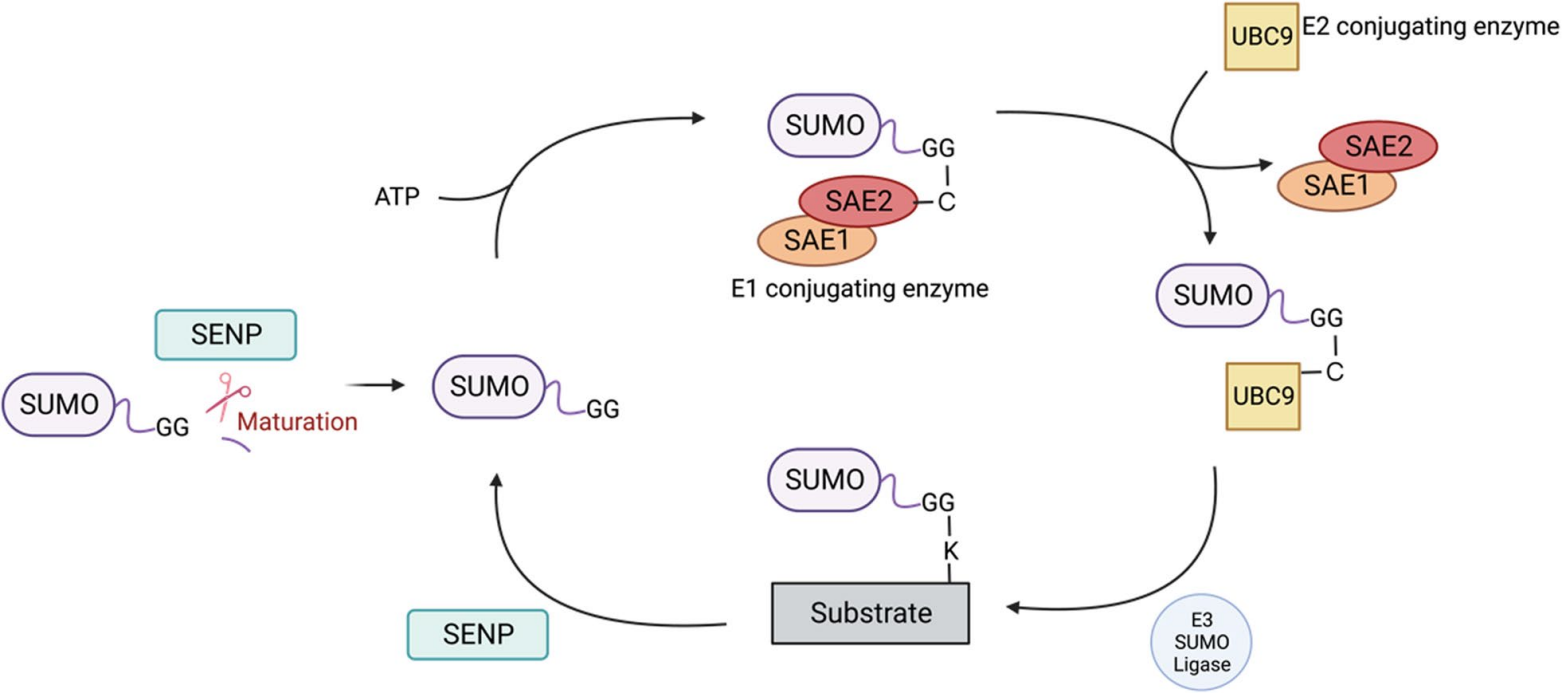
Model of SUMOylation. Initially, SUMO is an inactive precursor. SENPs, and sentrin-specific proteases catalyze and expose the diglycine (GG) motif of SUMO at the C-terminus. Then, through E1, E2, and E3 enzymes, SUMO is conjugated to the lysine (K) residue in the substrate that is often found in a consensus sequence. This modification modulates downstream biological functions of target proteins, such as protein–protein interactions and transcriptional regulation. SUMO attachment is reversible and removed from the substrate by SENPs

A series of enzymatic reactions (Fig. 1), including maturation, activation, ligation, and deconjugation, are required for SUMO conjugation [16]. Maturation of SUMO involves pre-cleavage at the C-terminus by SUMO-specific proteases (SENPs), which exposes the diglycine motif essential for SUMO ligation [9]. Then, SUMO activation requires SAE1/SAE2 heterodimer, an ATP-dependent E1-activating enzyme [17]. In the third step, Ubc9, the SUMO-conjugating E2 enzyme, receives SUMO from SAE1/SAE2, forms a thioester bond with SUMO and catalyzes covalent conjugation of SUMO with the substrate [18, 19]. Although Ubc9 is the primary E2 conjugation enzyme involved in SUMOylation, E3 SUMO ligases are usually required for efficient and specific conjugation of SUMO to target proteins. E3 ligases facilitate transfer of SUMO from Ubc9 to the lysine (K) residue of the target protein. There are several families of E3 SUMO ligases with different target substrates that regulate various aspects of protein functions [20–22]. Some substrates can be SUMOylated in an E3-independent manner, but such cases are generally rare [23, 24]. In E3-independent SUMOylation, the SUMOylation reaction is mediated primarily by Ubc9. However, in most cases, E3 ligases are critical to regulate the specificity and efficiency of SUMOylation [24–28]. There are three families of E3 ligases that have been discovered, the protein inhibitor of activated signal transducer and activator of transcription (PIAS) family [20, 29], RanBP2/Nup358 [22], and Polycomb member Pc2 [21]. They stabilize the interaction between the E2 enzyme and substrate protein, or facilitate orientating the target K residue [24]. SUMO-covalent binding to the K residue is often, although not always, embedded in a consensus sequence motif, ψ-K-X-D/E (ψ—large hydrophobic amino acid, X—any amino acid, D—aspartic acid, and E—glutamic acid), on the substrate [30, 31]. To be precise, three different mechanisms of K selection for SUMO conjugation have been demonstrated. First, Ubc9 directly binds to the K residue within the consensus site and catalyzes SUMO for conjugation. Second, target proteins can interact with the SUMO-interacting motif (SIM) in the SUMO moiety of Ubc9-SUMO thioester, which allows Ubc9 to catalyze the conjugation of SUMO with proximal K residues on the substrate. Lastly, E3 ligase mediates site selection by interacting with some target proteins with K residues, which involves the optimal positioning of Ubc9-SUMO thioester to catalyze the SUMO conjugation. In the latter two circumstances, the K residue does not necessarily have to be within the consensus site [32].

SUMO conjugation can be mono-SUMOylation, multi-SUMOylation, or poly-SUMOylation. Multi-SUMOylation occurs when SUMO targets multiple acceptor K residues within or outside the consensus site on the substrate. Poly-SUMOylation is mainly mediated by SUMO2/3 because internal SUMO consensus sequences enable iterative linkages of poly-SUMO chains on K11 [33]. At the end of the SUMOylation cycle, SUMO is removed from the substrate by SENPs.

**Fig. 2:**
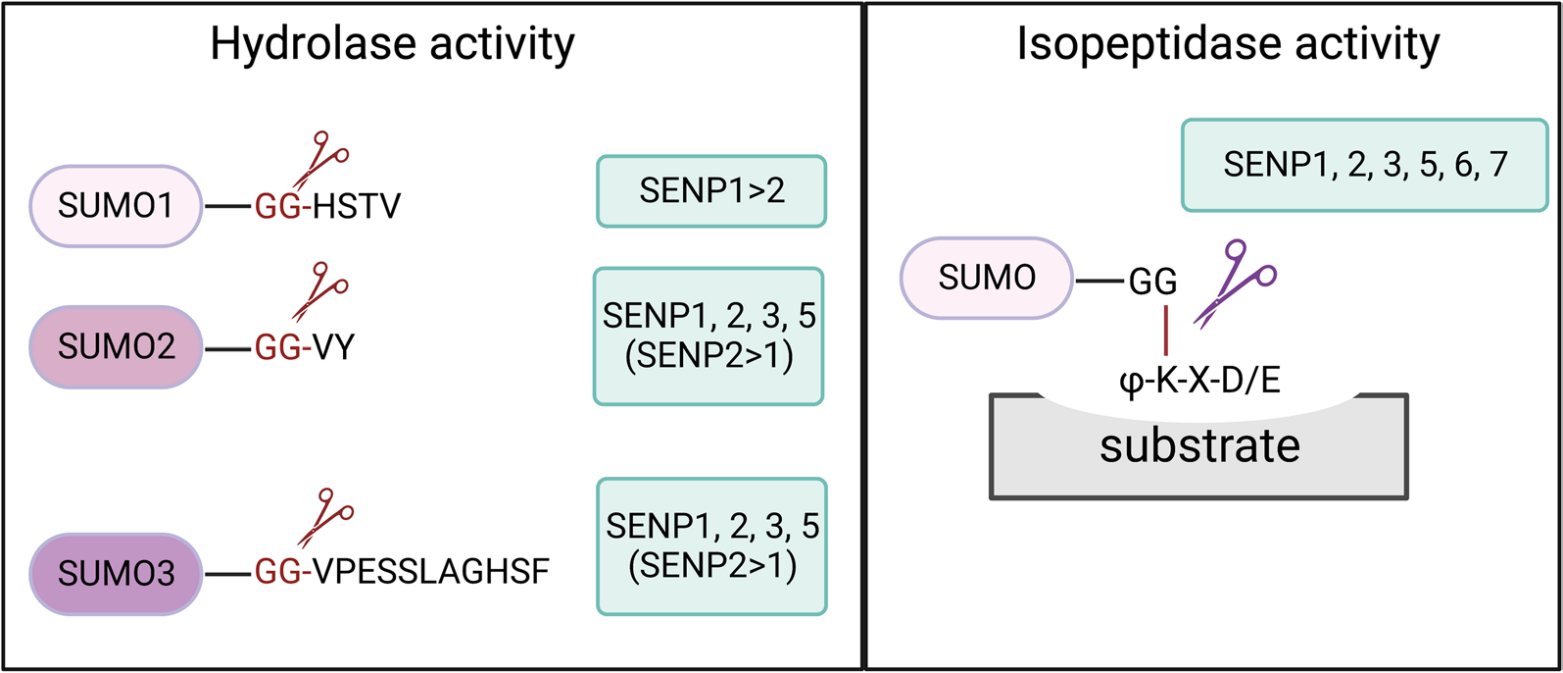
SENPs act in both maturation of SUMOs and deconjugation of SUMOs from modified proteins. Maturation of SUMO precursors relies on the hydrolase activity of SENPs (left). Different SENP family members have an inherent preference for maturation of different SUMO paralogs. However, the deconjugation of SUMO depends on the isopeptidase activity (right)

SENPs play a dual role in the SUMOylation process, functioning in both maturation and deconjugation steps. Maturation requires the hydrolase activity of SENPs to remove the diglycine motif from SUMO, whereas deconjugation requires the isopeptidase activity of SENPs to cleave the covalent bond between SUMO and the substrate protein (Fig. 2) [34].

Six SENPs exist in mammals, namely SENP1, SENP2, SENP3, SENP5, SENP6, and SENP7, all of which belong to the Ulp cysteine protease family [35]. The Ulp protease family has a highly conserved catalytic domain at the C-terminus [35, 36]. SENP1, SENP2, and SENP5 are involved in the maturation phase of SUMOylation with different preferences for SUMOs in vitro [14]. The deconjugation activities of SENPs are characterized by their different specificities. SENP1 and SENP2 remove all types of SUMOs with comparable efficiency, whereas SENP3 and SENP5 are more specific for SUMO2/3. SENP6 and SENP7 are only responsible for editing poly-SUMOylation chains mediated by SUMO2/3 [14, 34]. Different SENPs are found in subcellular locations depending on their N-terminal domains. For example, SENP1 is mainly located in the nucleus, and mutations in the N-terminal region of SENP1 cause cytoplasmic accumulation [37]. SENP3 resides primarily in nucleoli, mainly because of its interaction with the nucleolar scaffold protein NPM1 [38]. SENP2 is found at various locations in cells depending on its alternatively spliced forms. Full-length SENP2 is found primarily in nucleoplasmic nuclear pore complexes [36]. Another alternative spliced form of SENP2, SuPr-1, lacks an exon at the N-terminus and is enriched in PML bodies [39]. A murine homolog is Axam2 which lacks a different exon at the N-terminus and is found mainly in the cytoplasm [24, 40]. By combining the literature together, the N-terminus sequence of SENP2 appears to determine whether it is located in the cytoplasm, at nuclear pores, or in PML nuclear bodies [39–41]. Similar to SENP3, SENP5 is found in the nucleolus [42]. SENP6 and SENP7 are mainly found in nucleoplasm [43–45].

SUMOylation modulates the stability, localization, or activity of proteins, and interplays with the regulation by other PTMs, thereby affecting cellular processes and contributing to both homeostasis and disease states. SUMOylation acts synergistically, sequentially, or antagonistically with other PTMs including ubiquitination, phosphorylation, and acetylation [46, 47]. For example, poly-SUMO chains bridge further ubiquitination of common substrates via SUMO-targeted ubiquitin ligases, thereby allowing proteasomal degradation [48, 49]. Another report showed that SUMO itself is phosphorylated at threonine 76, which increases SUMO1 stability [50]. Furthermore, IκBα is ubiquitinated at K21 and subsequently degraded in the proteasome, leading to nuclear factor kappa B (NF-κB) activation. SUMOylation of K21 on IκBα provides protection against degradation. Ubiquitination of IκBα requires prior phosphorylation at serines 32 and 36, but phosphorylation of IκBα at both sites inhibits SUMOylation. Therefore, phosphorylation appears to antagonize SUMOylation [51]. Signal transducer and activator of transcription 5 (STAT5) is another example of modulating other types of PTMs [52]. Phosphorylation and acetylation of STAT5 are required for dimerization and translocation of STAT5 to the nucleus. Van Nguyen et al. showed that SUMOylation inhibits tyrosine phosphorylation of STAT5, changing STAT5 from an active to inactive state [52].

The discovery of phosphorylation-dependent SUMOylation motif (PDSM), ψ-K-X-D/E-X-X-S-P, is another example of the coordination of phosphorylation and SUMOylation [53–55]. PDSM contains a SUMO consensus site and a proline-directed phosphorylation site at the adjacent serine residue. It can be found in many transcription factors, such as myocyte-specific enhancer factor 2A (MEF2A), GATA-1, heat-shock factors (HSF1 and HSF4b) and PPARγ [53, 56]. Hietakangas et al. showed that the phosphorylation-dependent SUMOylation plays an important role in repressing the transactivation activity of HSF1 and HSF4b [53]. Mohideen et al. further showed that E2 Ubc9 is involved in the phosphorylation-dependent SUMO conjugation to MEF2A [54].

### General biological functions of SUMOylation

The importance of SUMOylation in mammals has been demonstrated in several reports [57–60]. Wang et al. showed that *Sumo1* knockout mice develop congenital heart defects and undergo premature death [61]. Wang et al. reported that *Sumo2*-deficient mice exhibit delayed development and die at the embryonic stage [62]. Moreover, Nacerddine et al. showed that embryos with a deleted *Ubc9* gene, the E2 enzyme required for SUMO1/2/3 conjugation, die at the early postimplantation stage because of the loss of nuclear integrity and chromosomal defects [58]. In terms of SENPs, Cheng et al. found that *Senp1* knockout mice exhibit severe fetal anemia because of deficient erythropoietin protein, leading to embryonic lethality [59]. Chiu et al. reported that *Senp2* knockout mice show abnormal cell cycle progression during trophoblast development and embryonic lethality [60]. These findings highlight the importance of SUMOylation for embryonic development.

### SUMOylation in diseases

Owing to the critical roles of SUMOs and SENPs in maintaining the balance of substrate proteins between SUMOylated and unSUMOylated states, it is conceivable that altered expression or abnormal functions of molecules in SUMO cycling can lead to various diseases. Indeed, some diseases, such as cancers, heart diseases, and autoimmune diseases, result from dysregulation of SUMOylation [3, 63–65].

In terms of SUMOylation in cancers, recent studies have revealed the significance of SUMOylation in the malignant properties of tumor cells and tumor immunology. For example, activated SUMOylation enables tumor cells to evade immunosurveillance by suppressing antigen presentation by the MHC-I pathway. Specifically, MYC overexpression in lymphoma increases SUMO2/3-conjugated SAFB (scaffold attachment factor B), a transcriptional corepressor that inhibits MHC-I gene expression [66]. However, blocking SUMOylation by TAK-981, a SUMOylation inhibitor that targets the E1 activating enzyme SAE2 [67], reverses the effect on MHC-I expression [68]. In cancer cells, SENP2 is a potential tumor suppressor because it negatively regulates the proliferation, invasion, and migration of osteosarcoma cells by promoting ubiquitination and degradation of SRY-box-9 (SOX9) [69], a transcription factor required during embryonic development [70]. Abnormal expression of SOX9 is involved in various cancers, such as osteosarcoma [71], lung cancer [72], and breast cancer [73, 74]. Additionally, SENP2 upregulates the migration of breast cancer cells and contributes to cancer stemness by regulating transforming growth factor (TGF)-β/Smad4-dependent signaling [75].

SUMOylation is also essential for cardiac functions and development by regulating various transcription factors [57]. For example, GATA4, a transcription factor crucial for cardiomyocyte differentiation [76], is modified by SUMO1 [77]. This modification augments the transcriptional activity of GATA4, which in turn increases the expression of cardiac genes such as *α-MHC* (α-myosin heavy chain) and *ANF* (atrial natriuretic factor) [77]. Disruption of the dynamic balance of SUMOylation and deSUMOylation cycling causes severe heart diseases [78]. As an example, SUMO1 modification maintains stability, ATPase activity, and calcium transient of SERCA2a, a calcium-transporting ATP2A2 ATPase, which is important for cardiac contractility. However, decreased expression of SUMO1-conjugated SERCA2a is often observed in patients and mouse models with heart failure [79]. Another example is SENP3, which plays an important role in vascular remodeling [80]. Cai et al. demonstrated that SENP3 is highly expressed in vascular smooth muscle cells during oxidative stress-induced vascular remodeling and is responsible for deSUMOylation of β-catenin (Wnt/β-catenin is involved in vascular remodeling [81]) to protect β-catenin from proteasomal degradation, suggesting a role of SUMO regulation in hypertension and atherosclerosis [80].

In the context of autoimmune disorders, SUMOylation of c-Maf is inversely correlated to disease severity in the NOD mouse model of type 1 diabetes. Specifically, the SUMOylation status of c-Maf is negatively associated with the level of IL-21 produced by T cells, which is involved in the pathogenesis of type 1 diabetes. SUMO-defective c-Maf promotes the generation of IL-21-secreting extrafollicular helper T cells and effector/memory CD8^+^ T cells [65]. These findings indicate that c-Maf SUMOylation has a regulatory role in diabetes pathogenesis. Moreover, accumulating evidence suggests that SUMOylation is involved in rheumatoid arthritis [82, 83]. Pascual et al. demonstrated that peroxisome proliferator-activated receptor-γ (PPAR-γ), which is a crucial regulator of anti-inflammatory responses in monocytes [84], macrophages [85], and fibroblast-like synovial cells [86], is modified by SUMO [85]. In their study using a macrophage-like cell line, the authors demonstrated that SUMOylation of PPAR-γ is facilitated by PIAS1 and required for its ability to target nuclear receptor corepressor-histone deacetylase-3 complexes on the promoter of proinflammatory genes [85], implying targeting PIAS1 for rheumatoid arthritis treatment [82].

Dysregulation of the development and differentiation of immune cells is linked to several types of diseases. Because SUMOylation dynamically regulates the functions of substrate proteins, we next focus on the functions of SUMOylation and the underlying effect on SUMO substrate proteins in immune cells and responses.

## SUMOylation in the immune system

### SUMOylation in lymphoid cell progenitors

Growing evidence suggests that SUMOylation plays an important role in regulating lymphoid cell development [2]. Previous studies have shown that SUMOylation is crucial for various aspects of lymphoid cell development. For example, Liu et al. reported that the E3 ligase of SUMO machinery, PIAS1, is essential for hematopoietic stem cell (HSC) maintenance [87]. PIAS1 plays a critical role in HSC self-renewal and prevents dormant HSCs from entering the active cycle. Additionally, PIAS1 regulates proper lymphoid cell differentiation by epigenetically repressing expression of *Gata1* [87], a crucial transcription factor in the myeloid-erythroid lineage [88]. Disruption of PIAS1 expression impairs lymphoid cell differentiation, particularly B cells. In particular, significant reduction has been observed in the expression of genes associated with early B cells, such as *Il7r* (interleukin-7 receptor subunit alpha), *Pax5* (paired box protein 5), *Ebf1* (early B cell factor 1), and *Igll1* (immunoglobulin lambda-like polypeptide 1), in *Pias1* knockout HSCs. However, T cell-related genes, such as *Gata3* (Gata-binding factor 3), are not altered. Thus, PIAS1 expressed in HSCs is essential to regulate HSC self-renewal and support B cell lineage differentiation [87]. Other reports have also shown the effect of disruption of SUMO cycling on the differentiation of lymphoid progenitors. For example, Van Nguyen et al. found that SENP1 is highly expressed in early B and T cells, and is crucial for proper lymphocyte development [52]. STAT5 is a downstream signaling molecule of IL-7R, which is essential for immune cell development and functions [89, 90]. Downstream of IL-7/IL-7R-mediated signaling, STAT5 activity is regulated by phosphorylation and acetylation [91, 92]. The phosphorylation and acetylation of STAT5 promote its dimerization and subsequent translocation to the nucleus to drive the transcription of target genes. Considering that K696 targeted by the acetyl group is the SUMO conjugation site of STAT5, SUMOylation prevents acetylation and converts STAT5 from an active to inactive form. SENP1-mediated removal of SUMO2 from STAT5 is crucial for STAT5 to re-enter the activation–inactivation cycle. An absence of SENP1 results in accumulation of SUMO2-modified STAT5 in early lymphoid precursors, which blocks STAT5 acetylation and subsequent signaling. These findings suggest that SENP1 plays a critical role in maintaining proper early lymphoid cell development by regulating STAT5 activity through deSUMOylation [52].

### SUMOylation in B cells

SUMOylation of death domain-associated protein (Daxx) plays a critical role in regulating the growth of early B cells. Daxx is induced by type I interferons (IFN-I) and interacts with Pax5 to guide Pax5 to be a transcriptional activator or repressor, thereby regulating B cell development [93]. Daxx contains a SUMO-interacting motif, which mediates the interaction with SUMO-conjugated proteins [94]. Daxx itself can be SUMOylated, and SUMO-conjugated Daxx suppresses the growth of early B cells. Specifically, SUMOylation of Daxx at K630 or K631 is important for its nuclear translocation in pro-B cells and required for by IFN-I-induced suppression of B cell development and apoptosis [95]. Dobreva et al. showed that the pre-B cell-specific nuclear matrix attachment region protein SATB2 is modified by SUMO1 or SUMO3, thereby reducing the expression of the immunoglobulin μ gene [96], in which PIAS1 enhances SATB2 SUMOylation. Matrix attachment regions are sequences crucial for chromatin organization and associate with the nuclear matrix, which affects transcriptional regulation [97]. Subnuclear localization of SATB2 is regulated by SUMOylation because SUMOylation of SATB2 is preferentially located in the nuclear periphery, explaining the reduced transcriptional activation [96].

In addition to the early stage of B cell development, SUMOylation participates in the regulation of functions of mature B cells, germinal center B cells, and plasma cells. SUMOylation regulates B cell receptor (BCR) signaling. In vitro studies conducted by Schmidt et al. showed that B cell-restricted factor B-cell regulator of IgH transcription (Bright) binds to the lipid raft of resting B cells. Following BCR ligation, Bright becomes conjugated to SUMO1 and disassociates from the lipid raft. The concentration of Bright in the lipid raft regulates the threshold of BCR signaling, i.e., less Bright bound to the lipid raft leads to stronger BCR signaling [98]. This study indicated that SUMO modification of Bright shapes the threshold of BCR signaling, implying the underlying relevance in immunological tolerance and autoimmunity. Thus, further studies are required to understand the importance of SUMO1-modified Bright in vivo. The functional significance of SUMO modification in other transcription factors important for the B cell lineage includes B lymphocyte-induced maturation protein-1 (Blimp-1), which is a master regulator of plasma cell differentiation [99]. Blimp-1 is modified by SUMO1 at the K816 residue, which is mediated by SUMO E3 ligase PIAS1 [100]. Blimp-1 interacts with chromatin modifiers such as histone deacetylase 2 (HDAC2) to repress target gene expression, leading to downregulation of the gene expression program of mature B cell identity, including *Pax5* and *Ciita*. However, SUMO conjugation-defective Blimp-1 (K816R), which carries a mutated SUMO acceptor site from K to arginine (R) at the 816 residue, interacts poorly with HDAC2. The decreased interaction between Blimp-1 and HDAC2 reduces the transcriptional repression activity of Blimp-1 and plasma cell differentiation. [100]. PIAS1 in B cells has also been implicated this process. Overexpression of PIAS1 in B cells leads to abnormal activation of MYC, which may contribute to the development of B cell lymphoma [101]. In terms of class switch recombination, TGF-β induces IgA class switching in B cells [102]. Smad4 is an intracellular signal transducer of TGF-β signaling [103]. SUMOylation of Smad4 promotes protein stability and nuclear localization of Smad4 [104]. Therefore, SUMO modification may regulate class switch recombination. However, overexpression of SUMO1, SUMO2, and SUMO3 does not affect TGF-β/Smad-mediated transcriptional responses of germline α in mouse B cell line CH12F3-2A [105]. Instead, E3 ligase PIASy and HDAC1 cooperatively inhibit TGF-β/Smad-mediated transcriptional responses of germline α. Transcription factor Yin Yang 1 (YY1) promotes long-distance DNA interactions and is required for class switch recombination [106]. PIASy-mediated SUMOylation of YY1 at the K288 site suppresses transcriptional activity of YY1 [107]. Therefore, it is plausible that SUMOylation regulates class switch recombination by modulating the function of YY1 [108].

### SUMOylation in T cells

Accumulating studies have indicated that disruption of SUMOylation machinery affects T cell development or functions. *Ubc9* deficiency causes defective T cell receptor (TCR)-driven cell proliferation, downregulates expression of activation molecules, such as CTLA4, PD-1, and ICOS, and reduces IL-10 production in regulatory T (Treg) cells, thereby compromising the suppressor function of Treg cells [109]. Ding et al. generated Treg-specific *Ubc9* knockout mice to show that conditional knockout of *Ubc9* in Treg cells resulted in defective TCR signaling and decreases in the stability and activity of the transcription factor interferon regulatory factor 4 (IRF4). IRF4 has been identified as the transcription factor responsible for Treg cells and plays an important role in generating Treg cells in peripheral lymphoid organs [110]. The expression of IRF4 is induced by TCR signaling. Mice with specific deletion of *Irf4* in Treg cells develop multiorgan autoimmunity due to exacerbated Th2 responses and plasma cell infiltration [111]. Therefore, SUMO regulates the function of IRF4 in Treg cells and immunotolerance. Ubc9 participates in T cell development, and T cell-specific knockout of *Ubc9* significantly reduces CD4 and CD8 single-positive T cell populations in the thymus and peripheral lymphoid tissues. Notably, *Ubc9* deficiency in T cells results in defective positive selection of thymocytes during transition from the double-positive stage to single-positive thymocytes. Additionally, less natural killer T (NKT) and Treg cells are observed in *Ubc9*-deficient mice than WT mice [112]. E3 ligase PIAS1 also plays a role in regulating T cell homeostasis. Natural Treg (nTreg) cells are critical to establish peripheral tolerance by self-antigen presentation and selection [113]. The frequencies of thymic and splenic nTreg cells increase significantly in Treg-specific *Pias1* knockout mice [114]. These findings suggest that PIAS1 negatively regulates nTreg differentiation.

As discussed, a *Senp1* knockout mouse study revealed that SENP1 deficiency results in embryonic lethality and severe defects in early T and B cell development [52]. SENP1 is highly expressed during the early stages of T cell development. *Senp1* deficiency results in accumulation of SUMOylated STAT5, which prevents STAT5 acetylation and signaling. Signal transduction of the IL-7-STAT5 cascade is critical for early T cell development in the thymus. Therefore, impaired STAT5 activation leads to severe defects in T cell development. Yang et al. showed that T cell-specific *Senp2* deletion in mice negatively regulates Th17 cell differentiation in the colitis mouse model and results in a more severe pathogenesis [115]. In particular, differentiation of non-classical Th17 cells (also called pathogenic Th17 cells), which are different from classical Th17 cells, is enhanced. Pathogenic Th17 cells differentiate from naïve T cells induced by TGF-β3/IL-6 or IL-6/IL-23/IL-1β-mediated signaling. Furthermore, pathogenic Th17 cells produce granulocyte macrophage-colony stimulating factor and IFN-γ, which is critical for the pathogenicity of Th17 cells in inflammation [116, 117]. Yang et al. further showed that SENP2 regulates nuclear translocation of Smad4, which promotes RORγt expression in Th17 cells, through deSUMOylating Smad4 at the K159 site. Additionally, SENP3 plays a role in maintenance of Treg cell stability. Yu et al. found that T or Treg cell-specific knockout of *Senp3* causes a global increase in SUMO-conjugated proteins, dysregulates immune tolerance, and ablates suppressor functions of Treg cells [118]. Furthermore, the authors found that *Senp3* regulates the functions of Treg cells by controlling the status of SUMOylation and nuclear localization of a repressor, BTB, and CNC homolog 2 (BACH2). BACH2 controls T cell maturation and differentiation [119]. Reactive oxygen species following TCR and CD28 stimulation induces SENP3 accumulation to regulate Treg cell stability.

Overexpression of SUMO2 in a T cell-specific manner in mice promotes differentiation of IL-17-producing CD8^+^ T cells with an efficient anti-tumor activity. Overexpression of SUMO2 in T cells suppresses tumor growth in vivo, linking to higher mRNA levels of IFN-γ and granzyme B in tumor tissues. Overexpression of SUMO2 also increases IL-6-dependent STAT3 phosphorylation [120]. These findings suggest that SUMO overexpression in T cells plays a role in anti-tumor responses.

The functions of several transcription factors are regulated by SUMOylation in T cells. Nuclear factor of activated T cells (NFAT) regulates the T cell proliferation and activation [121]. NFAT also regulates cytokine production and the expression of cytokine receptors [122]. Nayak et al. showed that an NFAT isoform, NFATc1/C, is highly SUMOylated, and its translocation is regulated by SUMOylation. SUMOylated NFATc1/C translocates to promyelocytic leukemia (PML) bodies in the nucleus, leading to the interaction of class I and II histone deacetylases (HDACs) and suppression of *Il2* expression [123]. Xiao et al. generated a transgenic mouse model, in which SUMO-sites of NFATc1, K702 and K914, are mutated. In their study, the authors found that the defect in NFATc1 SUMOylation ameliorates autoimmune and alloimmune responses in the disease model of experimental autoimmune encephalomyelitis (EAE) and graft-versus-host disease through the promotion of Treg cell expansion [64]. The authors showed that the increased IL-2 expression negatively regulates IL-17 and IFN-γ expression through the induction of STAT5 and Blimp-1. Downstream signaling molecules of TCR is also SUMOylated. For example, the activation of TCR signaling induces phosphorylation and activation of another transcription factor, JunB, which translocates into the nucleus and promotes the production of cytokines, such as IL-2, IL-4, and IL-10, in T cells [124]. JunB is SUMOylated at the K237 site. Blocking SUMOylation on JunB prevents expression of IL-2 and IL-4 in T cells [125]. Therefore, SUMOylation of JunB regulates its ability to induce cytokine expression for T cell activation.

SUMO modification modulates the function of transcription factors important for driving CD4^+^ T cell subset differentiation. For example, the transcription factor c-Maf is a Th2 cell-specific factor that transactivates the *Il4* gene [126] and is modified by SUMOylation at K33 [127]. Hsu et al. showed that SUMO modification of c-Maf regulates diabetes development through IL-21 signaling from CD4^+^ T cells in nonobese diabetic (NOD) mice. SUMO-defective c-Maf promotes IL-21 expression in T cells, and T cell-specific transgenic NOD mice overexpressing SUMOylation site-defective c-Maf resulted in more rapid development of the diabetes than control mice [65]. These data suggest that regulating the status of SUMOylation is an alternative approach to manage T cell-mediated inflammatory diseases. Mechanistically, SUMO-defective c-Maf selectively inhibits recruitment of HDAC2 to the *Il21* promoter, but enhances histone acetylation, mediated by cAMP response element-binding protein (CREB)-binding protein and p300, to transactivate *Il21*. Additionally, RORγt, a transcriptional factor critical for driving Th17 differentiation [128], is SUMOylated at K187 by Ubc9 [129]. SUMOylation of RORγt promotes binding of HDAC2 to the *Il17* promoter and inhibits IL-17 expression in Th17 cells. Mutation of the SUMO conjugation site K187 in RORγt facilitates disease progression in spontaneous colitis of the T cell transfer mouse model [130]. In the EAE autoimmune disease model, disruption of RORγt SUMOylation at the K31 residue downregulates Th17 differentiation and causes resistance to EAE induction [131]. RORγt is modified by SUMO3, which is catalyzed by the E3 ligase PIAS4 [131]. To mimic fever in humans, under the condition of febrile temperature, the transcription cofactor SMAD4 is modified by SUMOylation through Ubc9, which promotes Th17 differentiation and enhances disease progression in the mouse model of EAE [132].

### SUMOylation in dendritic cells and anti-viral responses

SUMOylation regulates the development and function of dendritic cells (DCs). Emerging evidence has shown that overexpression of SUMO may affect DC development and maturation. For example, Kim et al. showed that SUMO2 overexpression does not generally affect DC maturation, but shifts naïve CD4^+^ T cells to the Th2 type response in vitro [133]. Mechanistically, IκBα is modified by SUMO2, and ectopic expression of SUMO2 prevents translocation of NF-κB/p65 into nucleus, thereby reducing the binding of NF-κB/p65 to the *Il-12*/p40 promoter. In addition to SUMO2, IκBα is modified by SUMO1 and SUMO4, which is important for the stability of IκBα and translocation of the NF-κB subunit p65 [5, 51]. Therefore, all SUMO proteins appear to be able to regulate NF-κB activity in DCs.

In addition to its role in DC development and maturation, SUMO machinery affects the anti-viral responses of DCs. A splice variant of tripartite motif protein 5 (TRIM5α) protein is a retrovirus restriction factor in a species-specific manner [134]. An absence of TRIM5α restriction is observed in DCs derived from human and non-human primates. TRIM5α is modified by SUMO at K10, but disruption of the SUMO conjugation site does not affect the anti-viral activity of TRIM5α [135], suggesting that a non-covalent interaction with SUMO or SUMOylated proteins accounts for the anti-viral activity of TRIM5α. In DCs derived from humans and non-human primates, SUMOylated TRIM5α is imported into Cajal bodies, a type of nuclear body [136], in the nucleus, whereas deSUMOylation of TRIM5α causes its accumulation in Cajal bodies and nucleoplasm. Mechanistically, deSUMOylation of TRIM5α by the deSUMOylating enzyme USPL1 in Cajal bodies allows TRIM5α to be sequestered in the nucleus, leading to efficient type I interferon responses by the DNA sensor cGAS during retroviral infection. In support of this, treatment with SUMOylation inhibitor ginkgolic acid, which leads to cytoplasmic expression of TRIM5α, abrogates IFN-I responses and restores retroviral restriction. Therefore, the unique SUMO-dependent subcellular localization of TRIM5α in DCs accounts for regulation of retroviral restriction [137]. Another study also revealed the indispensable role of SUMO machinery in regulating the anti-viral function of DCs. Chang et al. found that the viral protein VP35 from Ebola Zaire virus interacts with PIAS1 and Ubc9, the main players in the SUMO modification cascade, to facilitate SUMOylation of IRF7 in DCs and macrophages. Ebola Zaire virus inhibits IFN-I responses, allowing for rapid viral replication, while not affecting proinflammatory cytokine production [138]. IRF7 is essential for IFN-I induction in plasmacytoid DCs [139]. VP35 promotes SUMO1- and SUMO3-mediated SUMOylation of IRF7 to inhibit *Ifn* gene transcription. In addition to IRF7, IRF3 is modified by SUMO, which inhibits interferon responses [140]. Further evidence supports the negative role of SUMOylation in regulating the anti-viral function of DCs. Decque et al. found that the absence of SUMOylation in DCs leads to a significant increase in IFN-β expression by regulating the distal element upstream of the *Ifnb1* promoter, resulting in enhanced resistance to viral infection [141]. Using *Ubc9* knockout bone marrow-derived DCs, the authors found that SUMOylation deficiency increases the production of proinflammatory cytokines and IFN-β. Additional evidence suggests that SUMO2/3 is crucial to suppress spontaneous IFN responses via a non-canonical pathway [142]. Knockdown of SUMO2/3 expression in THP-1 cells elevates expression of IFN-related genes, including *IFNB1*, *IFI27*, and *ISG15*. Together, these studies shed light on the potential relevance of SUMOylation in autoimmune disorders that exhibit abnormal type I IFN responses [142].

Additional evidence indicates that SUMOylation in DCs plays a significant role in anti-tumor responses. Under oxidative stress, SENP3 accumulates in the cytoplasm of tumor cells, which triggers deSUMOylation of IFI204 [143], a DNA sensor necessary for STING activation [143]. As a result, STING undergoes phosphorylation and activation. STING activation is critical to initiate the IFN-I response in DCs. Specific deletion of *Senp3* in DCs does not affect DC development, but promotes the growth of colon cancer tumor cells *in viv*o, which may be attributed to a defect in the cytosolic DNA-sensing pathway. The absence of SENP3 expression in DCs abrogates IFN-I responses, and the low frequency of IFN-γ-expressing CD4^+^ and CD8^+^ effector T cells in the tumor and draining lymphoid nodes results in poor anti-tumor activity [144]. However, PIAS1 and STAT3 collaboratively suppress the expression of iNOS in cytotoxic DCs, which is cytotoxic against tumor cells [145]. Therefore, the role of SUMO modification in DCs in diseases appears to be temporally regulated and likely depends on the stage of disease progression.

### SUMOylation in myeloid cells

Macrophages polarize to M1 or M2 subtypes. M1 macrophages have immunostimulatory properties and induce an anti-tumor immune response, whereas M2 macrophages have immunosuppressive properties and promote tumor growth [146]. Conjugating enzyme Ubc9 regulates macrophage polarization in prostate cancer and reverses the immunosuppressive effect of tumor-associated macrophages [147]. Xiao et al. found that macrophage-specific deletion of *Ubc9* in mice reduces the growth of prostate cancer cells in vivo. Tumor-bearing mice lacking *Ubc9* in macrophages have enhanced activation of macrophages and antigen-specific CD8^+^ T cells. Mechanistically, Ubc9-mediated STAT4 SUMOylation at the K350 site suppresses nuclear translocation and stability of STAT4 to affect the expression of IFN-γ and TNF-α. Wang et al. showed that SENP1 is responsible for KLF4 deSUMOylation and the SENP1-KLF4 axis participates in M1 macrophage polarization through the NF-κB signaling pathway [148]. Kruppel-like factor 4 (KLF4) is a transcription factor for macrophage polarization, and its SUMOylation is critical for macrophage M2 polarization [149]. KLF4 is SUMOylated at the K278 site. KLF4 SUMOylation-deficient macrophages promote the expression of M1 macrophage-associated genes in tumor cells and have strong anti-tumor activity. In the context of immunometabolism, macrophage polarization is associated with metabolic reprogramming. Glycolysis increases in M1 macrophages, whereas M2 macrophages have increased fatty acid oxidation and oxidative phosphorylation [150]. Sirt3 is a major deacetylase in mitochondria and regulates metabolic processes, including oxidative phosphorylation and fatty acid oxidation [151]. Zhou et al. showed that, upon stimulation by lipopolysaccharide (LPS) and IFN-γ, SENP1 enters mitochondria to deSUMOylate Sirt3, thereby activating Sirt3 to induce M2 macrophage polarization [152].

SUMO modification modulates therapeutic efficacy in certain myeloid cancers. Retinoic acid, a class of compounds derived from vitamin A, has been used for the treatment of acute promyelocytic leukemia (APL), a subtype of acute myeloid leukemia (AML) [153]. However, retinoid therapy has limited effectiveness in patients with non-APL AML. Baik et al. showed that inhibition of SUMOylation promotes the sensitivity of non-APL AML cells to all-*trans*-retinoic acid (ATRA) treatment [154]. Blocking SUMOylation by overexpressing either SENP2 or SENP5 makes non-APL AML cells more susceptible to ATRA and increases apoptosis. Therefore, inhibition of SUMOylation potentiates the anti-leukemic effect of ATRA. Additionally, Subasumstat, a small molecule inhibitor of SUMOylation, enhances the activity of rituximab, a monoclonal antibody used for treatment of B cell non-Hodgkin’s lymphoma [155]. Nakamura et al. showed that Subasumstat promotes IFN-I-dependent macrophage M1 polarization and macrophage phagocytosis. Subasumstat treatment also potentiates the activity of anti-CD20 monoclonal antibody (rituximab) in xenograft models [156]. These studies provide a strategy to improve the therapeutic efficacy in some cancer types by inhibiting SUMOylation.

SUMOylation in macrophages has been implicated in the regulation of inflammation. Activation of microglia, tissue macrophages of the central nerve system, during ischemic brain injury induces inflammation and tissue repair [157]. SUMOylation of annexin-A1 (ANXA1), which is involved in the resolution of inflammation, plays a crucial role in modulating microglial polarization after cerebral ischemia [158]. Specifically, SUMOylation of ANXA1 regulates the stability of IκB kinase, which inhibits NF-κB signaling [158]. SUMOylation of ANXA1 suppresses the activation of NF-κB in microglia. Overexpression of SUMOylated ANXA1 in microglia/macrophages improves neurological functions in a mouse model of cerebral ischemia. This study suggests that elevated SUMOylation of ANXA1 in microglia is another potential therapeutic strategy for stroke and neuroinflammatory diseases. Additionally, peroxisome proliferator-activated receptor gamma (PPARγ) suppresses the transcription of inflammatory response genes in macrophages through SUMOylation of PPARγ at the ligand-binding domain, which targets PPARγ to nuclear receptor corepressor-histone deacetylase-3 complexes and represses proinflammatory genes [85].

## Conclusions and perspectives

This review summarizes the current understanding of the SUMO-involved molecular and biochemical mechanisms. We also provided several examples of the roles of SUMO modification in health and diseases. Disruption of SUMO cycling causes a variety of cellular abnormalities and is relevant to various diseases. The significance of SUMOylation in various cell lineages, including immune cells, in physiological and pathophysiological states has prompted scientists to develop inhibitors to block or alter the cycling of SUMOylation. At least three categories of compounds/agents alter the SUMOylation process [159, 160]. One is SUMO mimics, which block endogenous SUMO from entering SUMOylation enzymatic cascades. Another category is enzyme inhibitors that target E1 or E2, or polypeptides that promote degradation of Ubc9 and PIAS1 E3. The other category is SENP inhibitors in the form of small molecule compounds or small hairpin RNA. These inhibitors, which are under development, have been tested in various diseases, including cancers, neurological disorders, human immunodeficiency virus infection, and cardiovascular diseases. Recently, small molecule TAK-981 was developed as a potent and selective inhibitor of the E1/SAE SUMO-activating enzyme [161], which is currently in phase I clinical trials for patients with solid tumors and lymphomas. TAK-981 induces IFN-I signaling and upregulates IFN receptor 1 in cells of the tumor microenvironment, thereby inhibiting tumor growth. The effect of TAK-981 on tumor growth is linked to increased T and natural killer cell infiltration and activation in tumors. Therefore, comprehensive studies of the biochemical mechanisms of SUMO regulation, the physiological role of molecules involved in regulation of SUMO cycling in cells, and the SUMO substrate proteins in particular physiological and pathophysiological states may facilitate the development of molecules to specifically alter SUMO machinery, which provides a new strategy to modulate disease progression.

## Data Availability

Not applicable.

## Abbreviations

APL: Acute promyelocytic leukemia
AML: Acute myeloid leukemia
ATRA: All-*trans*-retinoic acid
ANXA1: Annexin-A1
ATP: Adenosine triphosphate
BCR: B cell receptor
Bright: B-cell regulator of IgH transcription
Blimp-1: B lymphocyte-induced maturation protein-1
DC: Dendritic cell
Daxx: Death domain-associated protein
EAE: Experimental autoimmune encephalomyelitis
GATA: GATA binding protein
HDAC: Histone deacetylase
HSF: Heat-shock factor
HSC: Hematopoietic stem cell
IL: Interleukin
IFN: Interferon
IRF: Interferon regulatory factor
K: Lysine
KLF4: Kruppel-like factor 4
MEF2A: Myocyte-specific enhancer factor 2A
MHCI: Major histocompatibility complex I
NF-κΒ: Nuclear factor kappa-light-chain-enhancer of activated B cells
NFAT: Nuclear factor of activated T-cells
NOD: Nonobese diabetic
PTM: Post-translational modification
PML: Promyelocytic leukemia
PDSM: Phosphorylation-dependent SUMOylation motif
PPAR-γ: Proliferator-activated receptor-γ
Pax5: Paired box 5
PIAS: Protein inhibitor of activated signal transducer and activator of transcription
RORγ: Retinoic acid receptor-related orphan receptor γ
SUMO: Small ubiquitin-like modifier
SENP: Sentrin(SUMO)-specific proteases
SAE: SUMO-activating enzyme
SIM: SUMO-interacting motif
STAT: Signal transducer and activator of transcription
SERCA2a: Sarcoplasmic/endoplasmic reticulum Ca^2+^ ATPase 2a
SATB2: Special AT-rich sequence-binding protein 2
STING: Stimulator of interferon genes
Sirt3: Sirtuin-3
TGFβ: Transforming growth factor-beta
TCR: T cell receptor
Treg: T regulatory cell
Th: T helper cell
Trim5α: Tripate motif 5 alpha
Ubc9: Ubiquitin-conjugating enzyme 9
YY1: Yin Yang 1
